# Agriculture and Bioactives: Achieving Both Crop Yield and Phytochemicals

**DOI:** 10.3390/ijms14024203

**Published:** 2013-02-20

**Authors:** Lina García-Mier, Ramón G. Guevara-González, Víctor M. Mondragón-Olguín, Beatriz del Rocío Verduzco-Cuellar, Irineo Torres-Pacheco

**Affiliations:** 1 Biosystems Engineering Group, Biosystems Laboratory, Division of Graduate Studies, Faculty of Engineering, The Autonomous University of Queretaro, C.U Cerro de las Campanas, S/N, colonia Las Campanas, C.P. 76010, Santiago de Querétaro, Querétaro, Mexico; E-Mails: lgarcia37@alumnos.uaq.mx (L.G.-M.); ramon.guevara@uaq.mx (R.G.G.-G.); 2 Division of Graduate Studies, Faculty of Chemistry, The Autonomous University of Queretaro, C.U Cerro de las Campanas, S/N, colonia Las Campanas, C.P. 76010, Santiago de Querétaro, Querétaro, Mexico; E-Mail: vmmo_3@hotmail.com; 3 Division of Environmental Sciences and Technologies, School of Chemistry, The Autonomous University of Queretaro, C.U Cerro de las campanas, S/N, Col. Las Campanas, C.P. 76010, Santiago de Querétaro, Querétaro, Mexico; E-Mail: beatriz-verduzco@hotmail.com

**Keywords:** elicitor, organic agriculture, conventional agriculture, GMOs, secondary metabolite, plant stress

## Abstract

Plants are fundamental elements of the human diet, either as direct sources of nutrients or indirectly as feed for animals. During the past few years, the main goal of agriculture has been to increase yield in order to provide the food that is needed by a growing world population. As important as yield, but commonly forgotten in conventional agriculture, is to keep and, if it is possible, to increase the phytochemical content due to their health implications. Nowadays, it is necessary to go beyond this, reconciling yield and phytochemicals that, at first glance, might seem in conflict. This can be accomplished through reviewing food requirements, plant consumption with health implications, and farming methods. The aim of this work is to show how both yield and phytochemicals converge into a new vision of agricultural management in a framework of integrated agricultural practices.

## 1. Introduction

Food security is one of this century’s key global challenges. The world population will increase up to, at least, the middle of the 21st century, and demand for food will rise. Climate change also has a profound impact on food production. Furthermore, it remains a clear challenge to define ways in which agricultural production could contribute to improved health for all people [1,2].

Plants are essential for world feeding, not only by the nutrients they provide but also because they produce an enormous variety of secondary metabolites [3], such as phenolic compounds, terpenes, and alkaloids, with roles in various biological processes related to seed dispersal and resistance to stresses [4]. In order to feed a larger population agriculture has focused on intensive practices involving the use of improved crops, mechanical plowing, chemical fertilizers, and pesticides. Indeed, these practices derived from the Green Revolution technologies spread worldwide in the 1950s and 1960s, which significantly increased the yields produced per hectare of agricultural land [5]. However they have a negative environmental impact, emanating doubts if this is the right pathway that could face current challenges. Organic agriculture emerged with the perspective of producing safer foods with environmentally friendly techniques. Despite the healthy confidence due to better nutrient composition, organic agriculture constantly confronts doubts about its reliability to feed a growing population [6]. The necessary changes to global agriculture are not just a matter of quantity. In addition to increasing yield, there are further challenges concerning food quality, nutritional benefit, efficient management of plant pests and diseases, managing potentially adverse impacts, and reducing the environmental impact of technological change.

Agricultural improvements have usually focused on providing more “comfort” to plants to increase yield [7], but these measures have depressed the synthesis of phytochemicals because these products are usually produced during stressful situations [8]. On the one hand, the phytochemicals are needed by plants to defend themselves in a hostile environment, and on the other, are useful to herbivores like humans because they have beneficial effects on health [9]. For this reason, improvements in agricultural practices should focus not only on yield, but also on the maintenance and/or enhancement of phytochemicals present in plants.

Recent plant research focuses on exploring methods to induce secondary metabolites, which have been confirmed to possess many bioactive properties. Activated by plant defense systems, the production of secondary metabolites could reduce the use of pesticides. Chitosan, methyl jasmonate, and salicylic acid, among other elicitors, have been reported to be able to mimic biotic and abiotic stresses such as wounding, pathogen attack, UV light exposure, and plant temperature [8,10,11]. In most plants, stresses such as drought, irradiation, heat and salinity causes a variety of biochemical and physiological changes, which may affect plant metabolism, performance, and yield [12]. Consequently, these types of stresses have been also evaluated in crops during the application of elicitors.

Agriculture requires a shift in perspective with a particular emphasis on sustainability. Throughout this paper, the future world feeding needs, as well as the central role of plants in satisfying them, are presented; additionally, different agricultural practices are revised with special attention to the use of elicitors as a possible new element within integrated agricultural systems, and their effect on crop production and on the enhancement of phytochemical content to show that these substances could converge with yield.

## 2. Agricultural Systems

Agriculture has played a key role in the development of human civilization. It is an age-old activity, with origins in prehistory. When individuals formed clans, they started growing plants to guarantee an adequate food supply to meet their needs. When food production increased beyond the needs of a clan, food trade began. Over time, people became sedentary and the population grew. At that point it became necessary to accommodate plant needs in order to increase food production. Agricultural improvements to minimize plant stresses evolved until the advent of greenhouse automation, which accurately controls plant environment.

Nowadays, agriculture is still fundamental for the global food supply, and the changes around this issue are a response to different factors that deliver the need for structural change. As mentioned by Godfray *et al.* [13] issues for transitions in agriculture include gradual and sudden processes, like population pressure, changes in natural conditions, changes in markets and market prices, innovations, and applications of new technology. The direction of change in agriculture is determined by society (healthy food), economy, and environment.

Demands for an increase in food production were originally met by expanding the cultivated area. The scarcity of new land for crop production required an increase in crop production per unit area. This need for agricultural intensification has been commonly satisfied by the use of chemical fertilizers and improved cultivars. The application of synthetic fertilizers was the basis of the global increase in agricultural production after World War II, leading also to the practice of monoculture. Monoculture allows the field to be specialized toward producing maximum yield for a specific crop, but it is vulnerable to widespread outbreaks of diseases and pests. These agricultural practices [commonly known as conventional practices (CA)] were the result of the Green Revolution that took place between the 1940s and 1970s [14], and have made it possible that, compared to food consumption in 1961, each person today has (pro-rata) 25% more food [15].

Plant pathogens, pests, and weeds are responsible for a significant loss of potential global crop yield [7]. Feeding billions has been possible through the intensification of land and the use of pesticides. Similar to that of fertilizer, their use has increased all over the world; however this strategy has had an environmental cost and side effects such as eutrophication, agricultural dependence [16], and long term health effects [17]. Relationships between the use of pesticides and health diseases have been documented, such as organochlorine residues and breast cancer, PCBs, reduced sperm count and male sterility, birth defects, precocious puberty, and reproductive disorders [18]. In contrast with CA, and as a response to the Green Revolution, the concept of organic agriculture (OA) emerged as a way of low-input, or extensive farming [19]. The last decade has seen new developments in food production: organic agriculture and the genetic engineering of organisms.

Organic agriculture currently occupies 0.3% of agricultural land, mostly in developed countries [20]. Organic practices do not allow the use of chemical compounds for crop nutrition, synthetic compounds for pest, disease, and weed control; or the use of genetically modified (GM) cultivars. The acceptance of organic agriculture in developed countries is growing mainly because of environmental safety and health concerns. A significant reduction, of between 12% and 71% in most environmental impacts per hectare and year was reported by Nemecek *et al.* [21], when compared extensively with intensive farming management. In general, many authors reported that the environmental impacts are lower in organic agriculture [22,23]. Nevertheless, the products obtained organically cost more due to the careful practices needed in order to sustain the cultivars. Organic products could cost double than their conventional counterparts [24,25]. Additionally, the overall acceptance of OA is questioned due to its productivity, which it is said to be lower then compared with CA. This cast doubts upon the possibility to feed the entire world with OA [20]. According to Archer *et al.* [24], organic systems had lower corn yields, and generally lower wheat and alfalfa yields compared to the highest yielding conventional systems. However, soybean yields, for the highest yielding organic systems, were not significantly different from the highest yielding conventional systems. Nemecek *et al.* [19] also identified that the main drawbacks of organic farming for Swiss systems are their lower yields. De Ponti *et al.* [26] stated that organic yields of individual crops are, on average, 80% of conventional yields. Some growth plant parameters like leaf area and total plant dry matter are lower in organic practices when compared to conventional ones [27]. Chemical-free agriculture is gaining more and more support because of the idea of providing safer food, but it is still not able to respond to the need for producing massive amounts of food.

The perception among consumers is that organic cultivars possess a higher nutritional quality than conventionals (Figure S1). However, it is not easy to estimate compositional differences due to agricultural practices because of the great number of variables such as crop, irrigation patterns, weather variations, handling, *etc.* [28]. That is why the controversy remains whether or not organic foods have a nutritional and/or sensory advantage when compared to their conventionally produced counterparts. There is no agreement that one agricultural practice is more beneficial than another, since there are studies, which favor one, or another practice. For example, Huber *et al.* [29] indicate that a number of comparative studies showed higher levels of vitamin C, phenolic compounds, in organic plant products. Also, Pérez-López *et al.* [30] found that organic peppers had the higher content of minerals, carotenoids, and antioxidant activity when compare to conventional peppers. Kim *et al.* [31] found the same trend while Luthria *et al.* [32] concludes that multiple analyses become critical in order to draw any specific conclusions about the influence of environment and growing conditions on phenolic content. Moreover, some studies concluded that very few compositional differences exist. However it is stated that there is a higher nitrate content in conventionally produced vegetables in comparison with organic ones, possibly due to improper fertilizer applications [29,33]. This is relevant because high consumption of nitrates has been related with some cancers and methemoglobinemia [28].

Although organic systems required less purchased input, they need more fuel and labor, which results in higher total production costs compared to conventional production systems [24]. Besides, organic agriculture demands more land than conventional practices, and this land cost serves as an indicator of sustainability [34]. On the other hand, current methods of bulk production are reducing the nutritional quality content of the cultivars due to the high applications of inputs (especially fertilizers) to achieve faster growth [35].

Integrated crop management (ICM), a “third way” for agriculture between conventional and organic farming, has emerged over the past few years as a way to face the current agricultural challenges. The goals of ICM are to sustain agricultural production, maintain farm incomes, protect the environment, and respond to consumer concerns about food quality issues [36]. With this framework, the next two sections are going to summarize the current need of agriculture to respond to both crop yield and food with health benefits.

## 3. Achievement Crop Yield for Fulfillment of Global Food Requirements

As food is essential for survival and for mental and physical development, a minimum daily amount of calories is required; however, for populations at poverty level, obtaining food becomes an act of survival [37]. It is estimated that around 925 million people experience hunger; perhaps another billion are thought to suffer from “hidden hunger”, in which important micronutrients are missing from their diets. In contrast, a billion people over-consume, causing a new public health epidemic involving chronic diseases [38,39].

Issues of culture, economy, emotional comfort, religion, as well as advertising and availability, affect food production and demand, and also shape the market [40,41]. Furthermore, cooking and sharing meals are major social activities for many in middle- and high-income countries. Then, food production is topic of social and political stability for governments and other organizations [13,42].

The world food situation is being rapidly redefined by income growth, climate change, high energy prices, globalization, urbanization, and increased weeds, pests, and diseases [43]. These factors are transforming food consumption, production, and ultimately, markets [44]. The global food production has more than doubled during the second half of the 20th century in response to the doubling of the world population; however, the increase in food production has come at a cost, leaving a significant environmental footprint on the ecosystem. By 2050, world population will reach 9.1 billion and almost all of this population increase will occur in developing countries; it means a growth by 34% from 6.8 billion in 2009 [45], and a larger percentage, comparing with the more than 7.0 billion projected by the U.S. Census Bureau to August 2012 [46]. Life expectancy is also increasing; by 2045–2050 it is expected to have risen to 82 years in more developed regions, and to 75 years in lesser developed regions [47]. Urbanization will expand further, about 70% of the world’s population will be urban; also, income levels will be higher than they are now. In order to feed this larger, more urban, and richer population, food production must increase [48,49]. The increase of food production per capita could be obtained by expanding the area of agricultural land, enhancing the yield of crops and improving soil and water management; however not all are achievable: scarcity of water experienced in some places, as well as the trend to decrease current agricultural land [1,37]. Food producers are experiencing greater competition for land, water, and energy, and the need to reduce the negative environmental effects of food production is becoming an important topic [13].

According to Food and Agricultural Organization of the United Nations (FAO), global demand for food is expected to grow by 70% in the first half of this century [45], while crops may also be used for bioenergy and other industrial purposes [50]. New and current demand for agricultural products will exert pressure on already scarce agricultural resources. Also, it is important to mention that as income increases, lifestyles and consumption patterns change, resulting in diet diversification [51]. The demand for grains and other crops will decline, but vegetables, fruit, meat, dairy, and fish consumption will increase. In recent years, dietary guidelines have emphasized the consumption of wholegrain cereals, fruits, and vegetables due to their preventive effect on cardiovascular disease and other chronic diseases. This is relevant because it has been calculated that, in 2001, chronic diseases contributed approximately 60% of the 56.5 million total reported deaths worldwide [52]. This tendency has continued through the years; of the 57 million deaths that occurred globally in 2008, 36 million were due to chronic diseases. A large proportion of these deaths occurred before the age of 60, the most productive period of life. The magnitude of these diseases continues to rise, especially in low- and middle-income countries [53].

The primary elements of a diet are the three macronutrients: carbohydrates, protein, and fat. Awareness of the relationship between diet, health, and well-being has grown substantially in recent years. Additionally, people have shown a growing interest in food containing phytochemicals, commonly known as functional food, as a result of the increased evidence that a number of human health diseases are associated with diet and that specific food ingredients could have an impact on health [2,54]. Functional food is related to health promotion or disease prevention, and has acquired importance for both the general population and for policymakers due to the reduction in health care costs [55]. The global market of functional foods is estimated at 73 billion euros with an annual 8%–16% growth rate [56].

Currently the foremost issue is not only to supply food for everyone but also to improve the quality of that food. Beyond accomplishing global food production, it is important to consider several fundamental objectives of societies, including access to a healthy diet, reduction of malnutrition and poverty, better management of fresh water resources, and increased use of renewable energy. The goal is to produce more and better food for an increasingly demanding population and it should be achieved in a sustainable manner [13,57,58]. Although the yield using ICM tends to be lower when compared with conventional practices, it is considered that further research could reduce this gap [59]. For this reason ICM has been seen as a way to face the challenges stated above.

## 4. Phytochemical Enhancement for Health

In recent times, plants have been the focus of attention because of the claim is that “plants form the basis of the food web that sustains other forms of life” [58]. Fruits, vegetables, and whole grain cereals are foods that, the consumption of which, is currently encouraged by health authorities due to their phytochemical content [60,61]. Vegetables and fruits are dietary sources of one or more of the following nutraceuticals: vitamins C, B and carotenoids such as β-carotene and lycopene, polyphenols, flavonoids, folates, isothyocyanates, glucosinolates, and minerals [62,63]. Nevertheless, the content of these compounds is dependent on genotype/cultivar, growth condition, ripening stage, postharvest handling, and cooking conditions [32,55,64–67]. Conventional, organic, and integrated agricultural practices induce differences in the phytochemical content. It is reported that intregrated practices produce an intermediate phytochemical value between conventional and agricultural practices [30]; however it has been also presented that using integrated practices may be the same as with organic practices [68,69].

Plants are able to produce many chemical compounds through the process known as metabolism. Plant cells carry out both primary and secondary metabolism. Synthesis of substances necessary for the survival of the cells, such as polysaccharides, proteins, lipids, RNA, and DNA, takes place during primary metabolism through the use of aminoacids, sugars, fatty acids, and nucleotides. Secondary metabolism is activated only during a particular stage of growth and development, during periods of stress (biotic and abiotic) [9,70] or by the use of elicitors or signal molecules [71].

During secondary metabolism, plants produce thousands of phytochemicals, also known as secondary metabolites. These metabolites, are generally derived from primary metabolites through modification, such as methylation, hydroxylation, and glycosylation [72], were thought to be the result of aberrant metabolism and waste product of primary metabolism. They appear to have no direct growth and development functions. However, numerous studies have indicated that secondary metabolites in plants have well-established roles as protectors against predation, fungal and bacterial diseases, and against adverse climatic conditions [70]. In other words, they have an adaptive function related to the environment. This is a great advantage in a changing world. Secondary metabolites are important not only as plant defenders [73], but as signal molecules to attract pollinators and seed dispersers. It is important to mention that high concentrations of secondary metabolites might result in a more resistant plant; nevertheless, their production is usually considered costly and it reduces plant growth and reproduction [74,75].

Through their life cycle, plants face many adverse environmental conditions: drought, lack of nutrients, changing temperatures, and attack by pests and pathogens. However, despite the great amount of potential pathogens that might promote various diseases, plants are able to remain healthy because of defense mechanisms they have that enable them to adapt to different environments. These strategies can be classified into two types: physical and biochemical. Physical defenses are related to plant structure: e.g., the cell wall and cuticle. These two structures provide a mechanical barrier against pathogen penetration. On the other hand, biochemical defenses are related to the production of chemicals (phytoalexins, ROS, *etc.*) that are toxic for microorganisms. These toxic chemicals are produced when the plant recognizes the presence of a possible pathogen or the presence of elicitors, that is, chemicals from various sources that can trigger physiological and morphological responses on the targeted living organism, not only at the point of attack [76–78].

Plants use many resources in synthesizing secondary metabolites. They are produced through convoluted and regulated biosynthetic pathways operating in multiple cellular compartments. There is also evidence indicating that many biosynthetic pathways, leading to the accumulation of plant secondary metabolites, are not entirely active. Thus, unknown enzymes exist, sometimes without any apparent substrate or function, suggesting that plants have a reservoir of metabolic capabilities that normally remain hidden or unused [73]. To refer to these unknown metabolic capacities, Lewinsohn and Gijzen introduced the term “silent metabolism” [79]. It is estimated that a large quantity of identified metabolites is related to unrevealed enzyme functions because there is no correlation between the quantity of phytochemicals and their great diversity.

Plant defense mechanisms are characterized by signaling molecules crucial to regulate defense protein expression. Pathogens are able to evade multiple layers of defense. Therefore plants may respond by activating defense mechanisms that provide resistances to viruses, bacteria, fungi, nematodes, and insects. Salicylic acid (SA), jasmonic acid (JA), and/or ethylene (ET) are the major defense mechanisms identified [80,81]. The pathway related to salicylic acid can be prompted by pathogens, inducing systemic acquired resistance (SAR). The defense mechanism involving ET and JA provides resistance against necrotrophic fungi and insects. A third mechanism, also dependent on JA and ET, can be induced by rhizobacteria, creating the induction of systemic resistance (ISR). These defense mechanisms could be prompted by using elicitors that mimic the effects of different kinds of stresses, [71,82–84].

Secondary metabolites are more complex than primary metabolites. They are classified on the basis of chemical structure, composition, solubility in various solvents, or by the signaling pathway by which they are synthesized. They are commonly classified into terpenes (compounds whose composition is entirely carbon and hydrogen), phenolics (composed of simple sugar, benzene rings, hydrogen and oxygen), and alkaloids (composed of nitrogen or sulfur) [85]. Plants produce a diversity of these metabolites of which the mix is characteristic of each plant family, genus, and species [70]. Some compounds are restricted to individual species, others to related groups, and others still are only found in certain specific plant organs [74].

Although the presence of secondary metabolites in plants responds to an adaptive defensive system, recent concern for health-related, functional foods, has led to an emerging interest in the production of secondary metabolites due to their biological activities such as oxidative stress prevention, gene function regulation, and hormonal and immune modulation. In fact, current dietary recommendations suggest an increase in the consumption of foods that contain phytochemicals, since they provide beneficial effects to human health and play an important role in preventing chronic diseases, like cardiovascular disease, cancer, diabetes, *etc.* [86,87].

In recent years the relationship between fruit and vegetable consumption and health has been the main target of a great deal of scientific research involving the identification of specific plant components that promote health benefits. Bioactive compounds are classified into three major compounds classes of secondary metabolites found in plant-derived foods that may convey health benefits are exposed in Table 1.

## 5. An Approach to Sustainable Agriculture Using Elicitors

In the near future, food availability will be threatened if the right agriculture measures are not adopted. Nowadays, the reduction of pesticides to control pests and pathogens, and the presence of health compounds in food, is as important as food production. There is considerable agreement about the idea that increasing yields on existing agricultural land is a key component for minimizing further expansion [16]. On the other hand, it is known that plants have defensive mechanisms that are suitable to be used to develop sustainable agriculture production, taking into account not only a minimized use of pesticides but also an improvement in consumer’s health [108].

Plant defensive mechanisms could be encouraged through the use of elicitors [109,110]. In fact, it is known that treatment of plants with elicitors, or attack with pathogens, causes a set of defense reactions such as the accumulation of defensive secondary metabolites in edible and inedible parts of plants [70,82,108,111]. Some elicitors, such as Etephon, have either primary or secondary plant growth regulating action. The action of elicitors is similar to the one triggered by natural herbivore or pathogen infection [80]. It is well known that plants are sources of nutrient and phytochemicals. However, when treated with elicitors, they develop resistance to pathogens because application of elicitors on plant surface activates multiple signaling pathways of intracellular defense [80,112]. Elicitors can be classified according to their biological origin as biotic (polysaccharides, microorganisms, glycoproteins) or abiotic (temperature, fungicides, antibiotics, heavy metals, pH stress) [113,114]. Many substances have been discovered that work as elicitors [115]. Some examples are jasmonates [116], such as methyl jasmonate (MJ) [64,117] and jasmonic acid (JA) [111], other groups include salicylic acid (SA), benzothiadiazole (BTH), Etephon, hydrogen peroxide, and oligosaccharides such as chitosan [73,118], among other compounds. Actually, in the absence of pathogens, the use of an elicitor—like chitosan—has been seen to increase the seedling weight in tomato [112], and also to protect it from crown rot and root rot. In pearl millet, it has shown to reduce downy mildew [113]. Boonlertnirun *et al.* [119] indicates that chitosan is able to induce physiological and morphological responses that allow corn seedlings to survive under a hypoxic condition. Sprayed salicylic acid diminishes susceptibility to pathogens harm and abiotic stress, increases fruit tolerance to cold conditions, and increases storage life.

The effect of elicitors depends on many factors such as the concentration of the elicitor, time of elicitation, and stage in which elicitor is applied [73]. Also, elicitors can have a synergistic effect. Heredia and Cisneros Zevallos [64] reported that a combination of ET and MJ on wounded lettuce, celery, red onions, carrots, and jicama tissues amplifies the stress response possibly because both stresses may share common signaling molecules. Furthermore, the accumulation of secondary metabolites is influenced by the biosynthetic pathways activated in treated plants, depending on the compound used. For example, MJ modified the production of terpenoids in conifers [109], MJ increased the anthocyanin of apple fruit [116], and nitrogen and water stress resulted in higher flavonoid content [35]. The application of 200, 300 μM SA and 0.01% chitosan in five day old broccoli sprouts induced increases in its vitamin C content by 26%, 18% and 54%, respectively. Flavonoid concentration was also increased by 31% and 33% after 10 μM MeJA and 100 μM SA treatments, respectively in seven day old broccoli sprouts [120]. The application of ethyl acetate 0.05 M, MJ 0.001 and 0.005 M can be used at any growth stage to increase the total saponin content of soybean variety Ozark [121]. Puthusseri *et al.* [122] informed that foliar applications of salicylic acid (250 μM) in *Coriandrum sativum* L. enhanced folate levels twofold (3112.33 μg/100 g DW).

Depending on the dose, elicitors can cause phytotoxicity. For instance, Etephon affected plant growth form and had severe, dose-dependent, negative impacts on plant growth and flowering in tomato [80]. 2,6-Dichloroisonicotinic acid (INA) has shown broad-spectrum protection against pre-harvest diseases in many plants; however, its practical use is not favored due to a phytotoxicity problem [115]. Chen *et al.* [123] reported that application of MJ stimulated caffeoylputrescine accumulation in tomato leaves; however, high concentrations of the elicitor appeared to inhibit its formation. Compounds which might mimic the action of SA, such as *S*-methylbenzo [1,2,3] thiadiazole-7-carbothiate (acibenzolar-*S*-methyl) (ASM), have shown a reduction of mildew infection in wheat in field experiments, and they help to control infection by *Pseudomona syringae* pv. *tabaci*, *Cercospora nicotiana*, and *Alternaria alternate* in tobacco. Research has shown that β-aminobutyric acid (BABA) induces broad-spectrum resistance in a range of crops [113]. Application of the exogenous MJ increased the resistance of both the Aga and Kent strawberry cultivars to *T. urticae*, a serious pest of many fruits, vegetables, field crops, and ornamentals [124].

Elicitors act in many ways. One of them is through the oxidative burst which refers to the generation of reactive oxygen (ROS). ROS is an early part of the resistance mechanism of plant cells [125–127]. Also, after elicitation, the extracellular alkalization occurs as a result of the Ca^2+^ and proton influxes and the K^+^ efflux [128]. A single elicitor can lead to the activation of many defense genes. Despite the great interest on secondary metabolites, much of plant secondary metabolism is poorly understood.

**Figure 1:**
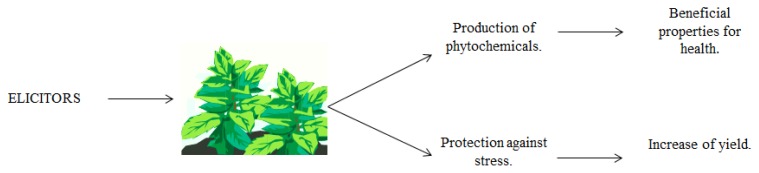
Application of elicitors as a sustainable perspective for agriculture.

As a regard to yield, Tierranegra-García *et al.* [129] reported that the application of SA and MeJA in 0.01 and 1 mM, and 10 and 100 mM did not significantly affect fresh or dry matter of roots, leaves, or total lettuce yields. Paradikovic *et al.* [130] reported an improved yield of 22% in peppers cultivars treated with commercial bioestimulants that contain elicitors when compared with controls. Benavides-Mendoza *et al.* [131] documented that the complex of poly (acrylic acid)-chitosan (PAA-Q) and benzoic acid (BA) induced yield increments when used at a concentration of 0.1% and 10^−4^ M in tomato, respectively. More recent findings indicated that the treatment of arable soils with rhizospheric microbes increases agronomic yields, as well as enhances the production of bioactive substances and protects them from pathogens [7]. These rhizospheric microbes are driving agents of nutrient cycling, regulating the dynamics of soil organic matter, soil carbon sequestration, and greenhouse gas emission; modifying soil physical structure and water regimes; and enhancing the efficiency of nutrient acquisition by the vegetation [132]. In a study carried out in open fields, bean crops were sprayed every 10 days with 0.05% chitosan. The control was sprayed only with water. It is indicated that treatments neither modified the phenological stages, nor altered the growth rate of bean plants, when compared to the control. Furthermore, chitosan induced a significant increase in the number of seeds and pods per plant [133]. Other works on sweet basil [134], and sunflower sprouts [135] established that chitosan treatments, either on plants or seeds, beyond improving growth and yield, enhanced qualitative qualities in terms of an increased synthesis of bioactive secondary metabolites, such as phenylpropanoids and isoprenoids. Bishnoi *et al.* [136] showed that in tomato, when Messenger® and Actigard®, two plant activators that contain elicitors, were used, yield increased from 10% to 13% in comparison to control. Boonlertnirun *et al.* [137] indicates that by applying chitosan to soil, and during seed soaking, the yield rice increased 17% in comparison to control. Figure 1 resumes the stated above.

To be sustainable, an activity must be viable from both an economic and an environmental point of view. Organic agriculture as well as integrated agricultural systems, with special focus to the use of elicitors, can contribute to sustainable food production systems; nevertheless, due to the lower yields of organic agriculture, the use of elicitors could become a viable strategy for the development of sustainable agriculture because, when they are applied to crops, a reduction in the use of agrochemicals can be achieved [138]. In addition, when they are applied at the right concentrations, the yields achieved with CA can be maintained, or even improved. Conrath establish that priming, using biotic or abiotic stress in plants, is important because it triggers their defenses mechanism; as well, because of the advantageous economic features [139]. This strategy represents an ecologically important adaptation to face environmental challenges. Nevertheless the use of these compounds requires further research in order to get the approval of more chemical elicitors for agricultural use, and overcome some of the disadvantages of their use, such as high cost of some elicitor, reduced plant fitness and growth, reduced photosyntesis, and pest disorientation [140–142]. Additionally, farm worker safety could be improved due to the low toxicity of elicitors [77]. Hence, the use of elicitors has well-established economic, social, and environmental implications.

## 6. Conclusions

As revised above, food requirements will increase in the upcoming years. In the meantime, per capita availability of arable land and irrigation water will decrease, year to year, while biotic and abiotic stresses expand. Farming systems should be considered in their entirety because every farming method has advantages and disadvantages, although it seems that elicitors are an approach to face the current challenges of agriculture. Elicitors can play an important role in the achievement of long-term crop productivity. Every day world population is increasing, not only in number, but also in life expectancy, so the production of high-quality food must increase with reduced inputs. This accomplishment is particularly challenging in the face of global environmental change. By carefully manipulating field-growing conditions through the use of elicitors, phytonutrients content can be maximized, improving human health as well as yield, while environmental impacts are reduced. The need for an increase in production in the near future, while conserving the resource base of agriculture, and minimizing adverse effects on the environment urges contributions from research in agriculture. Therefore priority has to be given to agricultural research related to elicitors in more crops to determine the most effective compounds, the right concentrations and application forms in order to improve the results of this practice.

## Figures and Tables

**Table 1:** Potential health benefits ascribe to three main classes of phytochemicals.

Active compounds	Potential health benefits	References
Polyphenols	Antiproliferative, antimutagenic, antioxidant, estrogenic, antimicrobial, anti-inflammatory, anticarcinogenic, cardioprotective, anti-itch, hypocholesterolemic, antidiabetic activity	[88–97]
Terpenes	Antioxidant activity, cancer prevention, cardioprotective activity, protection against eye diseases (cataracts, macular degeneration), antimicrobial, antidiabetic activity	[65,98–101]
Alkaloids	Antioxidant, antitumor, anticancer, anti-inflammatory activity, rheumatoid arthritis, hypertension	[102–107]

## Abbreviations

ROS: Reactive Oxigen Species
SA: Salicylic Acid
JA: Jasmonic Acid
ET: Ethylene
SAR: Systemic Acquired Resistance
ISR: Induction of Systemic Resistance
IPP: Isopentenyl Diphosphate
MEP: Methylerythritol Phosphate
OA: Organic Agriculture
PCBs: Polychlorinated Biphenyls
GM: Genetically Modified
GMOs: Genetically Modified Organisms
MJ: Methyl Jasmonate
BTH: Benzothiadiazole
INA: 2,6-Dichloroisonicotinic acid
ASM: *S*-methylbenzo[1,2,3] thiadiazole-7-carbothiate (Acibenzolar-*S*-Methyl)
BABA: β-aminobutyric acid
PAA-Q: Poly (Acrylic acid)-Chitosan
BA: Benzoic Acid
ICM: Integrated Crop Management

## Supplementary Files

Supplementary File 1 Supplementary Information (DOCX, 49 KB)
