# Supplementary material for: Agriculture and Bioactives: Achieving Both Crop Yield and Phytochemicals
Source: Int J Mol Sci. 2013 Feb 20;14(2):4203–22. doi: 10.3390/ijms14024203 (PMC3588095; doi:10.3390/ijms14024203)
Supplement: Supplementary File 1 — Supplementary Information (DOCX, 49 KB) [file ijms-14-04203-s001.docx]

Supplementary Information

**Figure S1.** Relationship between agricultural systems and production of phytochemicals in plant products.


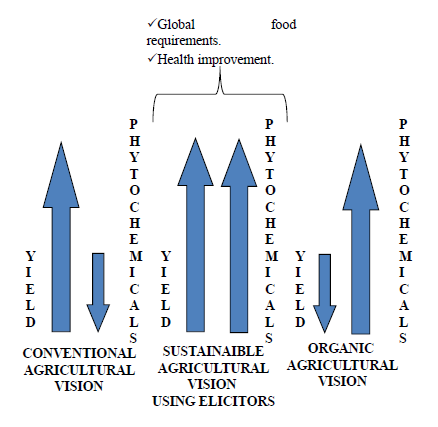


© 2013 by the authors; licensee MDPI, Basel, Switzerland. This article is an open access article distributed under the terms and conditions of the Creative Commons Attribution license (http://creativecommons.org/licenses/by/3.0/).
